# Eco-pilgrimages: Linking humans, heritage, and hydrology

**DOI:** 10.1007/s13280-025-02145-5

**Published:** 2025-02-28

**Authors:** Veronica Strang, Johannes M. Luetz

**Affiliations:** 1 School of Anthropology & Museum Ethnography, University of Oxford, 51/53 Banbury Road, Oxford, OX2 6PE UK; 2 Academy of Social Sciences, 33 Finsbury Square, London, EC2A 1AG UK; 3 Graduate Research School, Alphacrucis University College, Brisbane, QLD 4102 Australia; 4 School of Law and Society, The University of the Sunshine Coast, Maroochydore, QLD 4556 Australia; 5 School of Social Sciences, The University of New South Wales, Sydney, NSW 2052 Australia

**Keywords:** Aquatic ecosystems, Eco-pilgrimages, Heritage, Hydrology, Interdisciplinarity

## Abstract

Over the last century, the health of aquatic ecosystems around the world has reached critical levels. In the UK, waterways are severely polluted, and yet many wells and springs are still venerated as ‘sacred’. This article presents ‘eco-pilgrimages’ as a sustainability strategy to connect key heritage sites through ecological corridors. This aims, simultaneously, to strengthen biodiversity; to enable immersive historical and ecological education; to contribute to human well-being; and to provide more effective flood amelioration in river catchment areas.

## Celebrating water

**Fig. 1:**
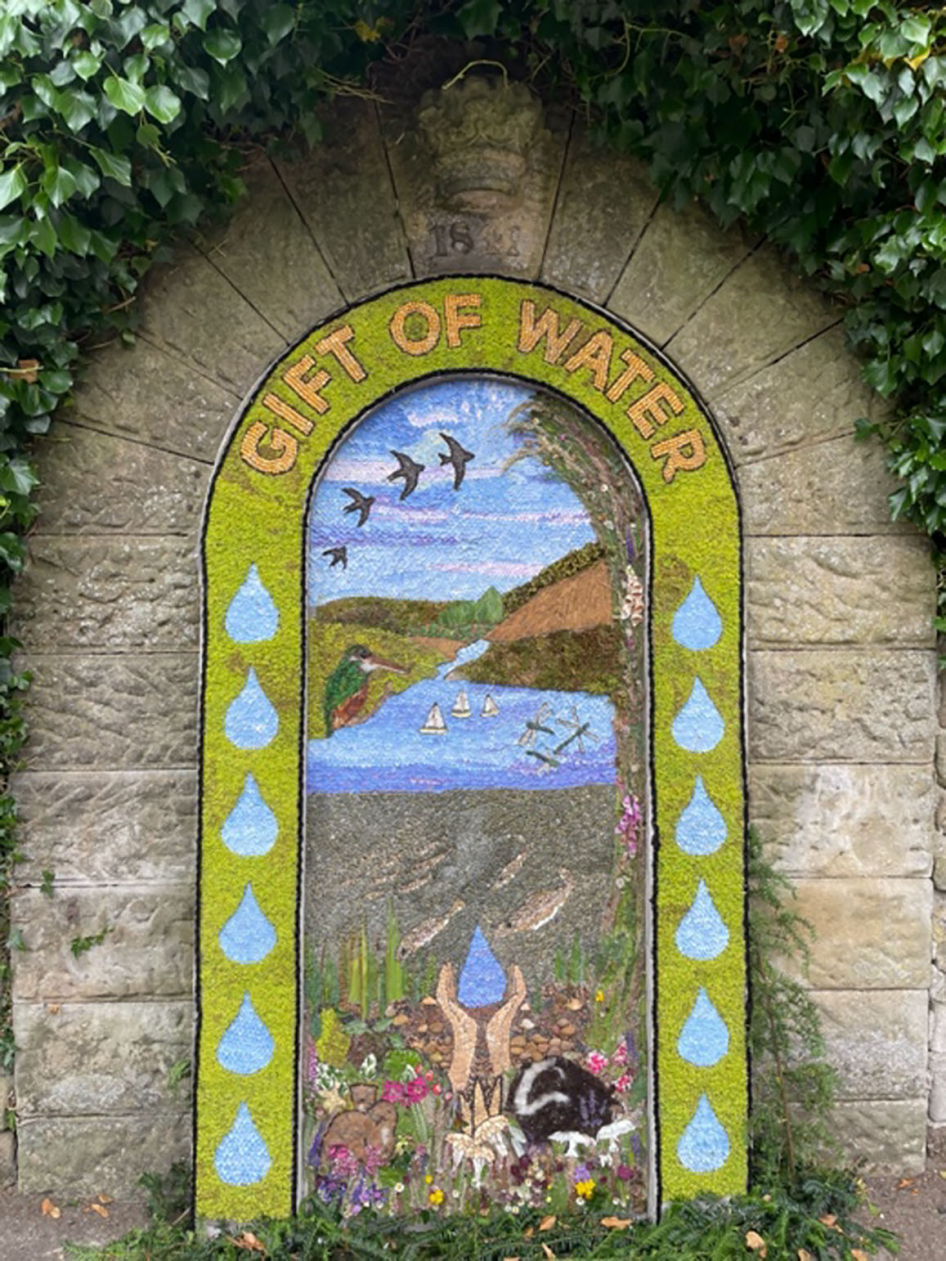
Well dressing at Rowsley Village, Derbyshire 2023. (Photo: Veronica Strang)

The benefits of spiritual pilgrimages and close engagement with the non-human domain have been amply documented in the literature but have not (yet) been brought together in the context of waterways in the UK. This approach responds to the pressing need for flood risk mitigation, made unequivocal by recent devastating flooding events across Europe (Piccolroaz 2024). Here, we explore a concept of ‘eco-pilgrimages’ utilising the historical veneration of water’s generative and healing powers while also supporting better river management. This article arose from discussions at a recent conference on *Sacred Waters*^1^ in Buxton, England, which brought together an international group of anthropologists, archaeologists, cultural heritage specialists, and artists to consider people’s interactions with ancient holy wells and springs (Ray 2020). Many of these focal water places are still celebrated, for example, through the well-dressings that take place across Derbyshire during the summer, in which local community groups decorate village wells with ephemeral artworks composed of flower petals, seeds, leaves, and other organic materials skilfully pressed into damp clay (Fig. 1).

**Fig. 2:**
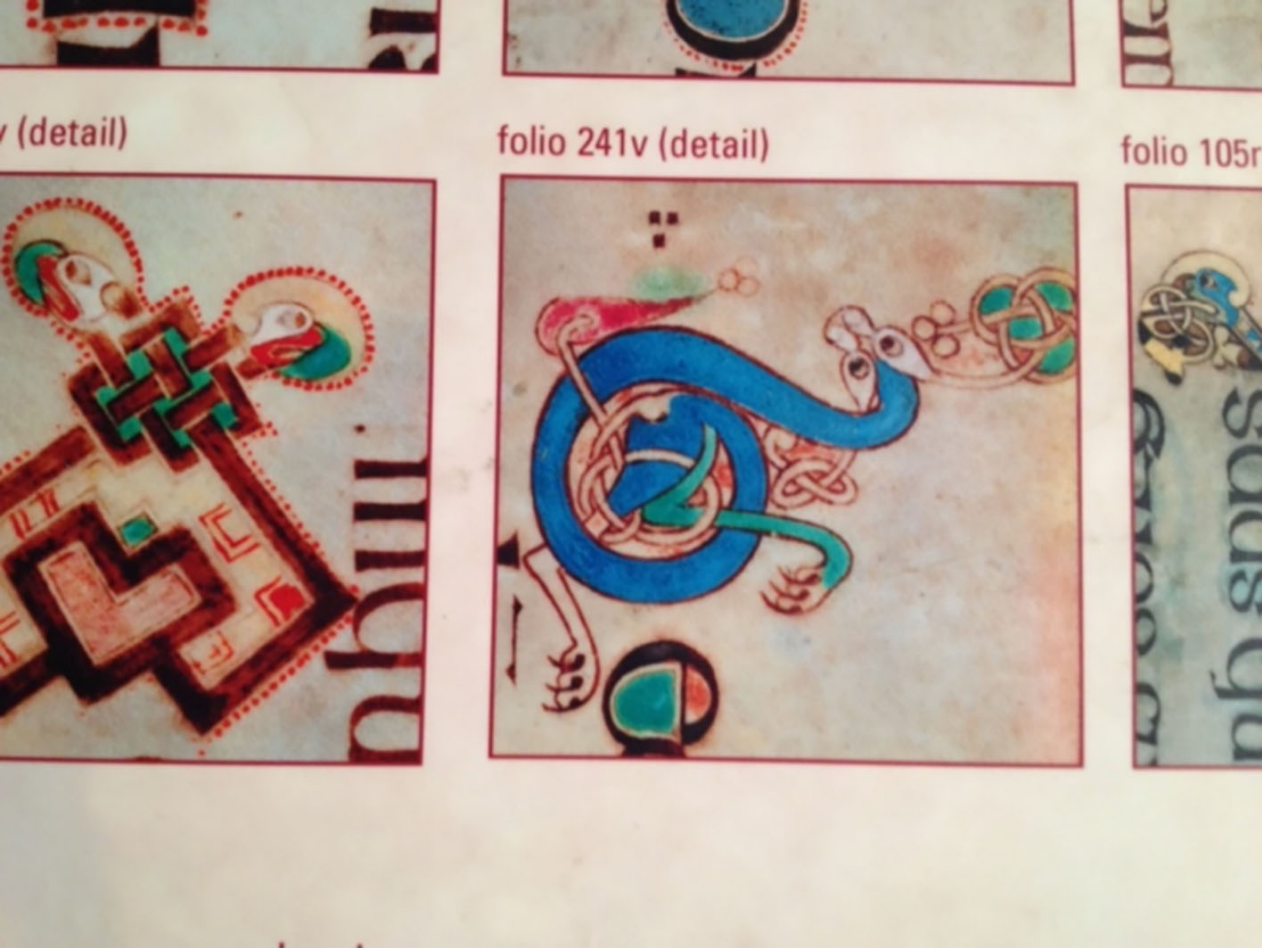
Detail from the Book of Kells, ca. 800 CE, Trinity College, Dublin. (Photo: Veronica Strang)

These annual celebrations recall much older European rituals at holy wells, such as Celtic votive offerings, and the Roman *fontanalia* that provides the etymological origin of church ‘fonts’. Such practices venerated water and the aquatic deities personifying its creative powers (Fig. 2), thus maintaining convivial relationships between humankind and the non-human domain (Strang 2023).

Today, many sacred water places are important bio-cultural heritage sites but, unless they are centrally located in towns and villages, they are often inaccessible and sometimes neglected. This is due in part to a lack of ready access to them and a related loss of knowledge about the human and non-human heritage that they embody. This raises a question as to how innovative policies might address pertinent challenges (and leverage opportunities) within the wider context of river catchment management.

The health of many waterways has been critically compromised in the last century. Essential riparian forests and wetland areas have been sacrificed to expand agricultural production, and the loss of these mediatory habitats has caused greater volatility in water flows and increased flood risks in downstream urban areas (Eriksen and Krause 2023). Farming chemicals and sewage continue to be discharged into rivers and streams, with their polluting effects sometimes exacerbated by the over-abstraction of water for human populations and irrigated farming (Bashir et al. 2020; Schürings et al. 2022). This combination of anthropogenic pressures has led to a radical loss of biodiversity in aquatic ecosystems globally, undermining the natural resilience of the biosphere and forming a key part of the emerging environmental crisis (Häder et al. 2021).

Still, there are some helpful success stories. In Dorset, for example, local conservation groups have worked with farmers and government agencies to establish the Stour Valley Way: a 64-mile footpath that gives walkers access to the entire length of the River Stour. In the process, farmers have been encouraged to re-establish riparian habitats, creating a connective ecological corridor from the source of the river to the coast, with commensurate gains in biodiversity. Although the Stour retains its natural tendencies to flood, these endeavours have gone some way towards steadying the hydrological flows of the river, reducing the risks to the housing that—as in so many river catchments—is located on flood meadows and around the estuary.

As this implies, human settlements have long been concentrated around waterways, and so too are many of the holy wells and springs that were historically vital to their flourishing. Whether or not we choose to imbue water with ideas about spiritual presence, sacred water places, such as those cherished in the Renaissance garden at Stour Head, serve as reminders that water and healthy aquatic ecosystems are literally essential to life, and must be better protected. So here is a vision for how this might be achieved.

## Eco-pilgrimages

There is no doubt that ecological corridors along waterways are highly supportive of biodiversity, with the added (and increasingly significant) benefit of restoring flood ameliorating habitats. But it is a challenge to re-arrange land use in this way. It requires real commitment from government and non-government bodies. It requires meaningful incentives to assist farmers and other landowners in sequestering riparian land and re-establishing biodiverse habitats. And each of these measures requires widespread public support.

In abstract terms, such support comes from ideological sympathy for changes in how we engage with the non-human world: for example, in the form of laws aiming to uphold non-human rights (Gray and Curry 2016); less anthropocentric governance and policy (Kopnina and Washington 2020), and greater appreciation of the reality that human and non-human well-being are interdependent and co-creative (Foley et al. 2019). More immediately, ‘on the ground’ changes depend on local communities having a shared understanding of the sometimes conflicting human and non-human needs and interests represented in river catchment areas. Much depends on people’s abilities to enjoy positive engagements with their local springs and waterways.

The anthropology of pilgrimage and tourism demonstrates the value of close engagement with the non-human domain, with manifold benefits for human mental health and well-being (Aerts et al. 2018). For some people, recreational activities such as hiking, birdwatching, canoeing, and wildlife observation foster a closer bond with local environments (Badone and Roseman 2004; Bradley 2009; Blau and Panagopoulos 2022). Others place a stronger emphasis on connecting with the non-human world in ways that they experience as spiritual, calming, and healing. But all positive interactions with aquatic ecosystems increase support for conservation and ‘greater experience with the natural environment [engenders] more pro-environmental attitudes’ (Hinds and Sparks 2008, p. 110).

**Fig. 3:**
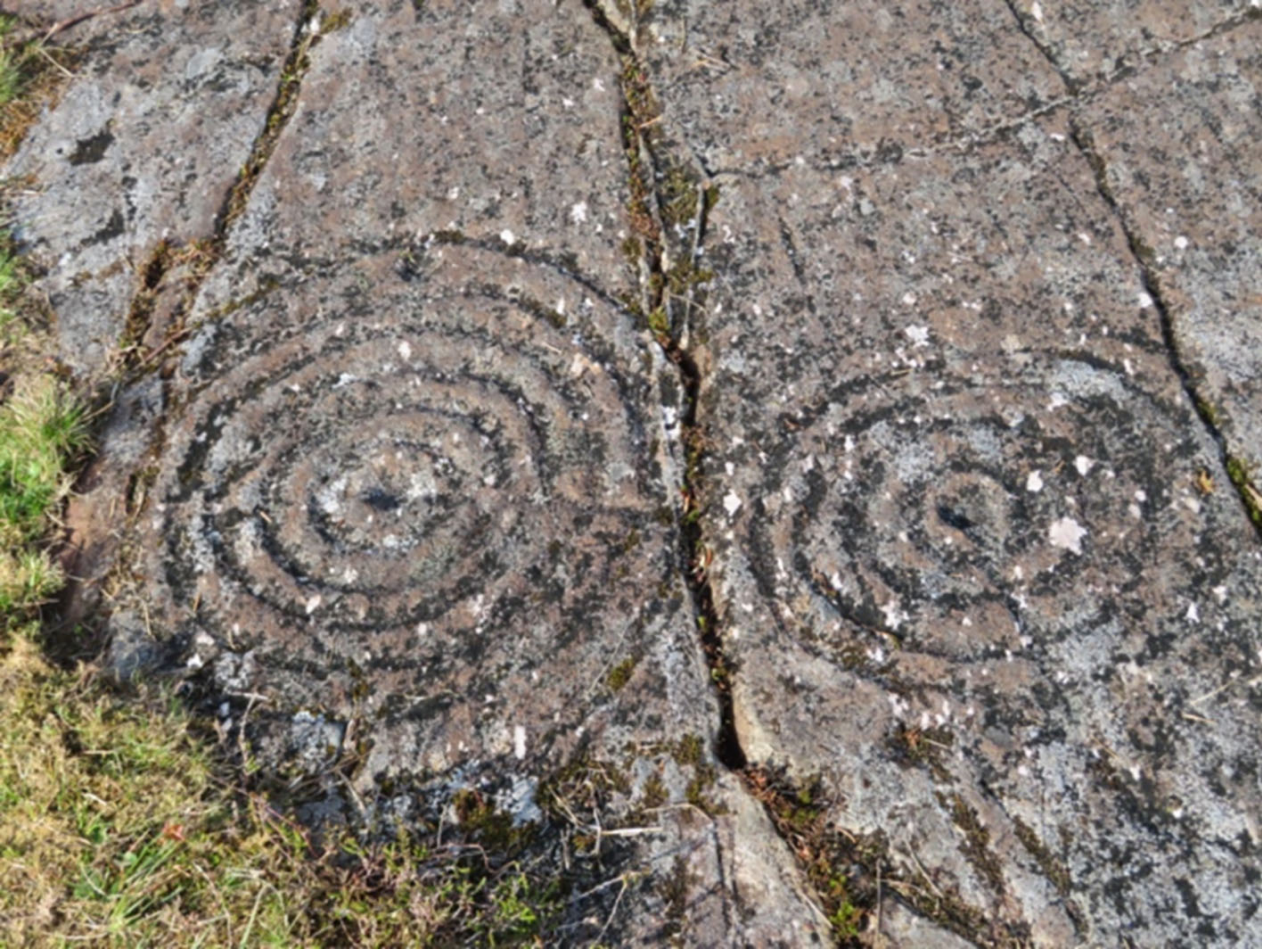
Ancient petroglyphs, Kilmartin Valley, Scotland. (Photo: Veronica Strang)

Convivial relationships with waterways are further encouraged by the provision of knowledge about them: their geology, their flora and fauna, and their bio-cultural heritage (Fig. 3). It is here that holy wells and springs, with their deep layers of archaeological and religious history, and their rich narratives about ancient water beings, have an important role in illuminating unique cultural waterscapes and providing contemporary populations with a sense of heritage, place, and belonging (Sy Su 2018).

## Local knowledge

Expanding communities’ knowledge about how water flows through local social and ecological environments can have important implications for climate change adaptation (Leal Filho et al. 2021). In effect, eco-pilgrimages can be pathways through which people can learn about their local aquatic ecosystems while at the same time becoming both more interested (and personally invested) in their conservation.

Highlighting the importance of cultural diversity, researchers in many parts of the world have noted the affinities between the traditional knowledge of place-based communities and environmental sustainability (Orlove et al. 2023; Luetz 2024). Local and/or Indigenous knowledge can inform sustainable water management in ways that are culturally meaningful and ecologically significant (Nelson and Shilling 2018; Borona 2020). So while this commentary proposes eco-pilgrimages in the broader context of waterscapes in the UK, there are similar opportunities in other countries. In Australia, for example, sacred sites abound along waterways. Indigenous eco-pilgrimage routes, based on traditional pathways, with the relevant communities providing educational insights into Aboriginal culture, could provide excellent opportunities to invite, recognise, and promote Indigenous knowledge and approaches to ecosystem conservation (Weir 2009; Lynch et al. 2013). Implementing joint endeavours to create eco-pilgrimages has the potential to offer both social and ecological gains, fostering collaborative relations between rural and urban communities, government agencies and conservation organisations, and supporting coherent agreement and action on ecological issues.

We suggest, therefore, that in many cultural contexts, there is a need for the systematic development of ecological corridors along springs and rivers, and that the government and non-government groups leading these changes should also think creatively about introducing ‘eco-pilgrimages’ connecting important heritage sites along the way, in particular those that reflect long traditions of venerating the generative and healing powers of water. Linking discrete heritage sites along waterways has the potential to transform them into vital corridors for conservation, education, and recreation.

Practically, this endeavour should come hand-in-hand with accessible and well-maintained pathways, providing ready access to user groups with diverse abilities and ensuring that eco-pilgrimages are inclusive and safe for all potential participants (Rossato and Baratta 2023). Alternate routes should be provided to disperse people sustainably across the landscape, thereby mitigating the environmental impacts of increased or fluctuating footfall and ensuring that conservation goals are prioritised (Park et al. 2008; Coppes and Braunisch 2013). The provision of well-organised and affordable public transport along eco-pilgrimage routes is equally important, both to ensure equitable access to the countryside and to avoid increases in car traffic.

By foregrounding the cultural meanings of water, and the history of human interactions with it, eco-pilgrimages have the potential to deepen participants’ appreciation of how waterways connect and support all living kinds. Such journeys would surely assist our collective efforts to protect river catchments and the well-being of their human and non-human communities.

## Data Availability

The authors declare that all data sources supporting this article are available within the paper.

## Footnotes

1 Our article owes much to the lively exchanges of ideas at the *Sacred Waters* conference in Buxton, 30 June–3 July 2024, organised by Celeste Ray, Peter Hewitt, and David Petts; https://sacredwaters7.wordpress.com/.
